# Automated and semi-automated 3D echocardiographic software for aortic annulus sizing in transcatheter aortic valve implantation helps bridge the gap between expert and novice operators

**DOI:** 10.1007/s10554-023-02885-z

**Published:** 2023-08-12

**Authors:** Charles Massie, Martine Parent, Maxime Berthelot-Richer, Rémi Kouz, Donald Palisaitis, Viet Le, Frédéric Poulin

**Affiliations:** 1 Hôpital du Sacré-Cœur de Montréal, 5400 Boul Gouin O, Montreal, QC H4J 1C5 Canada; 2 Hôtel Dieu de Québec, Quebec, QC Canada

**Keywords:** Three-dimensional echocardiography, TAVI, Aortic annulus, Automated, Novice

## Abstract

3D-transesophageal echocardiography (3D-TEE) is an alternative to multidetector row computed tomography (MDCT) for aortic annulus (AoA) sizing in preparation for Transcatheter aortic valve implantation (TAVI). We aim to evaluate how the fully automated (auto) and semi-automated (SA) TEE methods perform compared to conventional manual TEE method and the gold standard MDCT for annulus sizing both in expert and novice operators. In this prospective cohort study, eighty-nine patients with severe aortic stenosis underwent multimodality imaging with 3D-TEE and MDCT. Annular measurements were collected by expert echocardiographers using 3D auto, SA and manual methods and compared to MDCT. A novice in the field of echocardiography retrospectively measured the AoA for all patients using the same methods. TEE measurements, independently of the method used, had good to very good agreement to MDCT. They significantly underestimated aortic annular area and circumference vs. MDCT with the auto method underestimating it the most and the manual method the least (6.5% and 1.3% respectively for area and circumference). For experts, the manual TEE method offered the least systematic bias while the SA method had narrower limits of agreement (LOA). For the novice operator, SA method provided the least bias and narrower LOA vs. MDCT. There is good agreement between novice and experts for all 3 TEE methods but better agreement with auto and SA methods as opposed to manual one. Our study supports the use of 3D-TEE as a complementary method to MDCT for aortic annular sizing. The newer auto and SA software, that requires minimal operator intervention, is an easy to use, reliable and reproducible tool for aortic annulus sizing for experienced operators, and especially less experienced ones.

**Supplementary Information:**

The online version contains supplementary material available at 10.1007/s10554-023-02885-z.

## Introduction

Transcatheter aortic valve implantation (TAVI) has become an alternative to surgical aortic valve replacement in patients with high [1], intermediate [2, 3] and low surgical risk [4]. The accurate sizing of the aortic annulus (AoA) is essential in preparation for TAVI. An undersized prosthesis increases the risk of paravalvular regurgitation (PVR) [5] and valve migration, while an oversized prosthesis can lead to potentially fatal aortic injury such as periaortic hematomas [6] or aortic rupture and increases the risk of permanent pacemaker implantation. Importantly, moderate to severe post-procedural PVR has been associated with increased mortality [7]. Three-dimensional modalities have shown superior to two-dimensional modalities in predicting PVR and determining the choice of valve prosthesis [8]. Multidetector row computed tomography (MDCT) is currently the gold standard for AoA sizing but is associated with constraints related to administration of iodinated contrast, exposure to radiation and presence of artefacts (mainly motion artefacts) [9]. A manual measure of the AoA using 3D-transesophageal echocardiography (TEE) is used as a complementary method for AoA sizing but can lead to both over- and underestimation of the AoA area (AAA) [10, 11]. A novel echocardiographic software is now capable of generating dedicated automated (auto) and semi-automated (SA) measures of the AoA. Recent data show good correlation and acceptable bias between TEE and MDCT measures using the SA model of the software [12], as well as other similar software [13, 14]. A gap remains however as AoA size underestimation with TEE makes it a less reliable tool for prosthesis selection, with some authors suggesting a correction factor with TEE-based measures [12]. With the rapid expansion of TAVI indications and volumes, less experienced centers might be confronted with performing preoperative TEE. While agreement and correlation of the SA method vs. MDCT have been studied previously [12], its performance when used by less experienced operators has yet to be established and the fully automated model has not been validated yet. We hypothesized that the newer fully automated method, with minimal input from the operator and no manual editing, will help close the gaps between TEE and MDCT but also between novice and expert readers.

To do so, we aimed:

1. To compare measures of AoA performed by auto, SA, manual TEE methods and MDCT, the latter being used as gold standard.
2. To compare measures of AoA obtained by novice vs expert operators using all 3 methods.
3. To evaluate reproducibility of auto, SA, and manual TEE methods.

## Materials and methods

We conducted a prospective single-center cohort study. Consecutive patients with severe symptomatic aortic stenosis undergoing workup for TAVI with either balloon-expandable (Edwards SAPIEN 3) or self-expandable (Medtronic Evolut R or Evolut Pro) valves between November 2016 and December 2020 were eligible for inclusion at Hôpital Sacré Coeur de Montréal, an intermediate-volume TAVI center in Montreal, Quebec. Excluded patients had either not undergone all imaging modalities or images were not interpretable (i.e., artefacts, no valve section). Patients with a prior bioprosthetic aortic valve were also excluded. The study was approved by the ethics committee and consent was obtained for all participants.

### 3D-Echocardiography

#### Automated and semi-automated methods

Routine pre-procedural 3D-TEE was performed on all patients in the echo lab prior to the procedure by experienced echocardiographers. In our study, experienced echocardiographers are attending echocardiographers (FP and VL) and echocardiography fellow (MP). Once images were acquired from a mid-esophageal position they were transferred on an external workstation (EchoPAC) where analyses were completed using the commercially available software 4D Auto Aortic Valve Quantification (AVQ) (GE, Vivid E95), which is a dedicated software for AoA sizing.

**Fig. 1:**
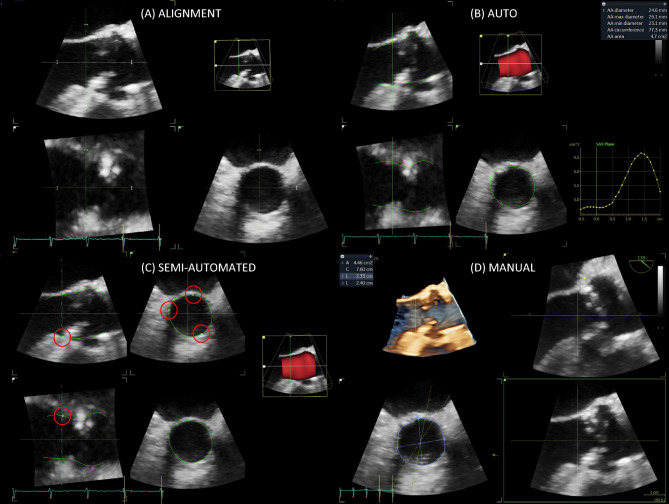
3D-TEE auto, SA and manual methods (**a**) Automatic alignment of the left ventricular outflow tract (LVOT). The 3 orthogonal planes are then manually translated to intercept the leaflet hinge points. **b** Automated LVOT segmentation and generation of auto AoA measurements. **c** Manual adjustments (red cercles) can be made to the software generated borders to better delineate the blood-tissue interface. The generated measurements (not shown here) are said to be semi-automated. **d** Manual contouring using the turnaround technique described by Kasel et al.[9] results in manual AoA measurements *(left upper corner)*

The automated 4D AVQ method allows the operator to perform computer assisted alignment and segmentation of the left ventricular outflow tract (LVOT). To do so, the operator must first choose the appropriate loop to perform measures on, taking care to avoid artefacts as feasible. The alignment of the LVOT is done automatically in mid-systole presenting the user with 3 orthogonal planes, two long axis slices and a short axis view of the annulus plane, that need to be adjusted successively to delineate the exact level of the annulus (Fig. 1a). Once that is done, the LVOT is automatically “segmented” (i.e., the annular contour is automatically drawn) and the software generates dimensions of the AoA[12]. These measurements include mid-systolic AAA, circumference, minimal (Dmin) and maximal diameters (Dmax) and mean diameter (Dmean) (Fig. 1b). Alternatively, once LVOT segmentation is completed, further manual adjustments can be performed by the user if deemed necessary (Fig. 1c). Both the values generated automatically by the software (fully automated or auto) and obtained after manual adjustment (semi-automated or SA) were collected. Only images with a minimum volume rate of 13 frames per second and minimal stitching and reverberation artifacts were used for measurements regardless of the underlying cardiac rhythm. The echocardiographer was blinded to the results obtained using the manual TEE method and MDCT.

#### Manual method

Using the images from the same pre-procedural 3D-TEE, measures of the AoA were obtained according to a standard protocol for manual 3D image acquisition described by Kasel et al.[9] (multiplanar reformatting with 3D volumes done on GE EchoPAC using the flexi-slice module). Mid-systolic AAA, circumference, Dmax, Dmin and Dmean were collected (Fig. 1d). The echocardiographer was blinded to the results obtained by auto and SA methods as well as MDCT.

### MDCT

All patients underwent MDCT prior to the procedure. Sizing of the aortic annulus was done at mid-systole by a single experienced operator (RK) with the use of 3mensio medical software (3mensio Medical Imaging BV, Bilthofen, The Netherlands), according to the technique described by Jilaihawi et al. [15] and Kasel et al.[9]

### Prosthesis selection

MDCT-derived AAA was used for valve size selection in balloon expandable valves, while MDCT-derived AoA circumference (or perimeter) was used for valve size selection of self-expandable valves, as per manufacturer-generated algorithms [9, 16, 17]. While measures obtained by both MDCT and 3D-TEE were taken into consideration by TAVI team, the final decision for prosthesis sizing was based primarily on MDCT measurements which is the current reference standard.

### Novice measurements

A novice in the field of echocardiography (CM) retrospectively measured the AoA for all patients using auto, SA, and manual methods. The novice had completed between 1 to 3 years of post-graduate core internal medicine training at the time of data collection with no formal TEE training. The methodology on the EchoPAC software was reviewed and 10 cases were performed under expert supervision prior to starting measurements. The novice was blinded to the measures previously obtained by the experienced operators.

### Outcomes

All outcomes were adjudicated according to the standardized Valve Academic Research Consortium 2 (VARC-2) guidelines [18] by 2 cardiologists. Clinical data were collected prospectively from systematic follow-up of all patients. Echocardiographic evaluation was performed by 2 experienced cardiologists in accordance with published guidelines from the American Society of Echocardiography [19].

### Post TAVI transthoracic echocardiography (TTE)

Echocardiographic evaluation was performed approximately 1 month after TAVI by 2 experienced cardiologists in accordance with published guidelines from the American Society of Echocardiography [19]. PVR was defined according to VARC-2 recommendations [18].

### Statistical analysis

Baseline, clinical, para-clinical, and procedural characteristics were expressed as means with standard deviation (SD) for continuous variables or as counts with percentages, as appropriate. The different imaging methods were compared using systematic bias analysis and correlation. Agreement between methods was evaluated using Bland Altman plots. Average bias was defined as mean difference ± SD and limits of agreement (LOA) were defined as ± 1.96 SD. Correlation was assessed using Pearson correlation coefficients and using the bootstrap resampling method (1000 samples) to determine the 95% confidence intervals (CI) (bias-corrected and accelerated method). Bootstrapping is an efficient approach to assess average model performance (internal validation) in small cohorts [20].

The intra- and interobserver variability were evaluated with the intraclass correlation coefficient (ICC). All statistical analyses were conducted using SPSS, version 28 (IBM SPSS Statistics for Windows, Armonk, NY: IBM Corp). A two-tailed p-value < 0.05 was considered statistically significant.

## Results

From November 2016 to December 2020, 89 consecutive patients with severe symptomatic aortic stenosis underwent multimodality imaging with 3D-TEE and MDCT in preparation for TAVI. Baseline clinical and echocardiographic characteristics are presented in Table 1. Of those, 19 patients did not undergo TAVI for multiple reasons (including decision of surgical aortic valve replacement, and conservative management) but were still included in the analysis. The incidence of significant PVR was low in our study with 94% of patients having mild PVR or less at 1 month post procedure. Since the TAVI procedural data was not available in all patients, this exploratory analysis was limited to a subgroup of 70 patients and can be found in the Supplemental appendix (Table S1). AAA and circumference derived from the different imaging methods are presented in Table 2 while measures and statistical analyses for Dmean, Dmax and Dmin can be found in the Supplemental Appendix.

**Table 1:** Baseline clinical and echocardiographic characteristics (n = 89)

Clinical characteristics	
Age, years	81 ± 6.5
Male sex	48 (53)
Obesity	33 (37)
Diabetes	25 (28)
Hypertension	76 (85)
PAD	12 (13)
Smoker	8 (9)
History of MI	10 (11)
History of PCI	10 (11)
History of CABG	13 (15)
Prior stroke	11 (12)
Atrial fibrillation	19 (21)
Anticoagulation	19 (21)
Chronic renal insufficiency†	36 (40)
Creatinine, μmol/L	92 ± 28
Glomerular filtration rate, mL/min/1.73 m^2^	63 ± 17
STS score (%)	3.9 ± 2
NYHA	2.4 ± 0.6
Aortic valve calcium score	2729 ± 1684

Echocardiographic characteristics	
LVEF, %	60 ± 11
Vmax, m/s	4.1 ± 0.7
Mean aortic gradient, mmHg	44 ± 11
AVA, cm^2^	0.8 ± 0.2
Aortic regurgitation	
None	18 (23)
Trace	32 (41)
Mild	19 (24)
Moderate	9 (11)
Severe	1 (1)
LVOT, mm	21.8 ± 2
Bicuspid aortic valve	4 (5)
Aortic valvuloplasty	4 (5)
Type of valve implanted (n = 70)	
Edwards S3	42 (60)
Evolut R	22 (31)
Evolut Pro	6 (9)
Vascular access	
Transfemoral	68 (97)
Transaxillary	2 (3)

Values are mean ± SD or frequencies (percentage)

*PAD*: peripheral artery disease; *MI*: myocardial infarction; *PCI*: percutaneous coronary intervention; *CABG*: coronary artery bypass graft; *STS score*: Society of Thoracic Surgeons score; *NYHA*: New York Heart Association; *LVEF*: left ventricular ejection fraction; *Vmax*: maximal aortic velocity; *AVA*: aortic valvular area; *LVOT*: left ventricular outflow tract

^†^ Glomerular filtration rate < 60 mL/min/1.73 m^2^

**Table 2:** Aortic annular measurements derived from different imaging methods (n = 89)

	EXPERT			NOVICE			MDCT
	Auto	Semi-automated	Manual	Auto	Semi-automated	Manual	
AAA (cm^2^)	4.1 ± 0.9*	4.3 ± 0.9*	4.3 ± 0.9*	4.1 ± 0.9*	4.3 ± 0.9*	4.3 ± 0.9*	4.6 ± 1.0
Circumference (mm)	72.3 ± 7.9*	73.2 ± 7.6*	76.0 ± 8.0	72.1 ± 8.1*	73.7 ± 8.0*	74.5 ± 8.4*†	77.0 ± 8.1

*AAA*: aortic annular area; *Auto*: automated method, *MDCT*: multidetector row computed tomography

*p < 0.001 for the comparisons between the TEE measurement vs. the reference MDCT

^†^p < 0.05 for the comparisons between the TEE measurement by novice vs. expert

### (A) Expert TEE vs. MDCT

All three TEE methods (auto, SA and manual) underestimated AAA and circumference, as compared to MDCT (Table 2). The auto method underestimated AAA and circumference respectively by 10.9% and 6.1% vs. 6.5% and 4.9% for SA method vs. 6.5% and 1.3% for the manual method (*data not shown)*. Of note, there was no statistically significant difference for AoA circumference between manual TEE method and MDCT.

#### Systematic bias

**Fig. 2:**
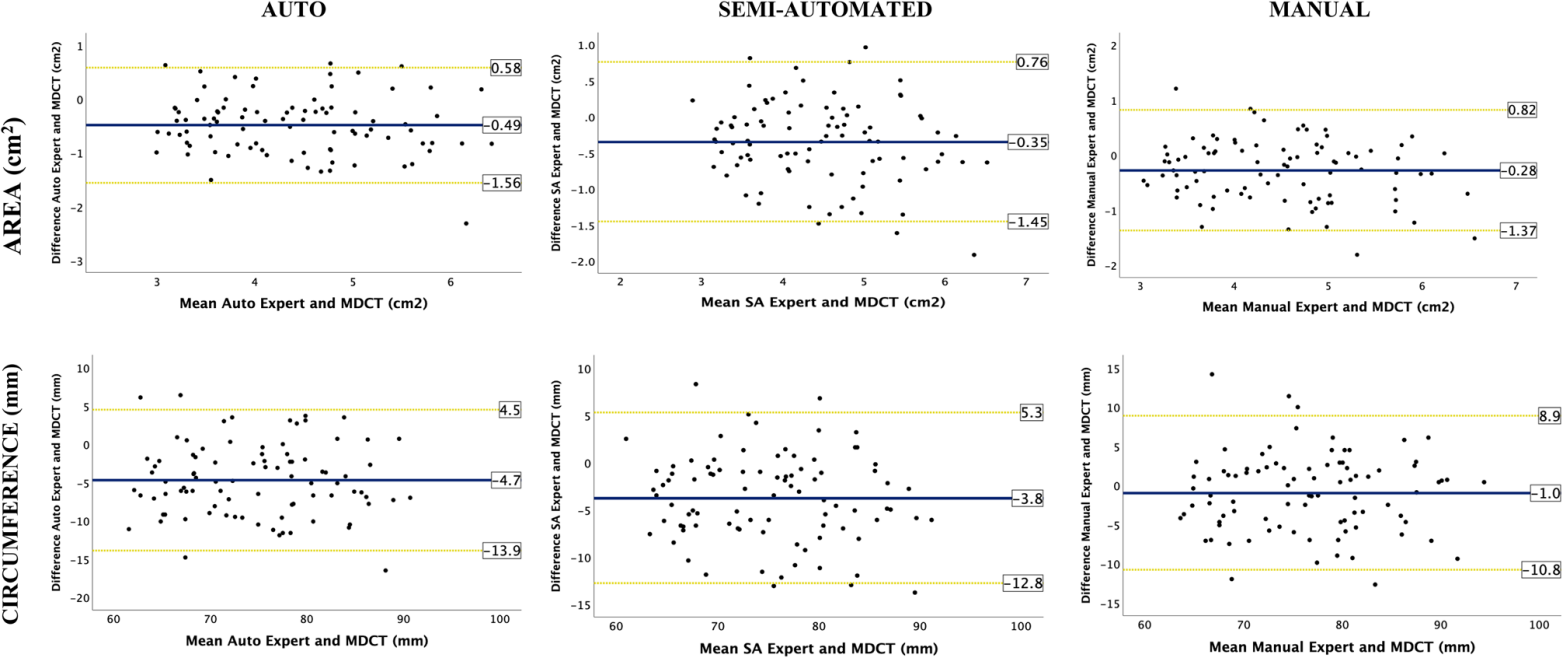
TEE expert vs. MDCT The Bland–Altman plots for aortic annular area (AAA) and circumference by automated, semi-automated and manual methods done by experts compared to MDCT

For the expert operators, good agreement was found between all three TEE methods and MDCT (Table 3). The manual method had the smallest systematic bias compared to MDCT, but SA and auto methods offered narrower limits of agreement (LOA) respectively for circumference and AAA (Table 3, Fig. 2). The auto method numerically underestimated MDCT dimensions the most (Tables 2 and 3).

**Table 3:** Average bias and LOA between modalities—AAA and circumference

**TEE EXPERT vs. MDCT**						
	Auto		SA		Manual	
	MD ± SD	± 1.96 SD	MD ± SD	± 1.96 SD	MD ± SD	± 1.96 SD
AAA (cm^2^)	− 0.49 ± 0.55	2.14	− 0.35 ± 0.57	2.22	− 0.28 ± 0.56	2.19
Circumference (mm)	− 4.7 ± 4.7	18.4	− 3.8 ± 4.7	18.2	− 1.0 ± 5.0	19.7

**TEE NOVICE vs. MDCT**						
	Auto		SA		Manual	
	MD ± SD	± 1.96 SD	MD ± SD	± 1.96 SD	MD ± SD	± 1.96 SD
AAA (cm^2^)	− 0.47 ± 0.56	2.19	− 0.27 ± 0.50	1.98	− 0.34 ± 0.52	2.06
Circumference (mm)	− 4.9 ± 4.7	18.5	− 3.3 ± 4.4	17.1	− 2.5 ± 5.6	21.8

**TEE NOVICE vs. EXPERT**						
	Auto		SA		Manual	
	MD ± SD	± 1.96 SD	MD ± SD	± 1.96 SD	MD ± SD	± 1.96 SD
AAA (cm^2^)	0.01 ± 0.40	1.58	0.07 ± 0.47	1.83	− 0.06 ± 0.55	2.14
Circumference (mm)	− 0.2 ± 3.7	14.4	0.5 ± 4.1	16.1	− 1.5 ± 6.2	24.5

*LOA*: limits of agreement; *AAA*: aortic annular area; *MD*: mean difference; *SD*: standard deviation; *Auto*: automated method; *SA*: semi-automated method

#### Correlation

All TEE methods had very good correlation with MDCT with regards to AAA and circumference (r between 0.81 and 0.83) (Table S4).

### (B) Novice TEE vs. MDCT

For the novice, all three TEE methods (auto, SA and manual) also significantly underestimated AAA and circumference, as compared to MDCT (Table 2). The auto method underestimated AAA and circumference the most, respectively by 10.9% and 6.4% (data not shown).

#### Systematic bias

**Fig. 3:**
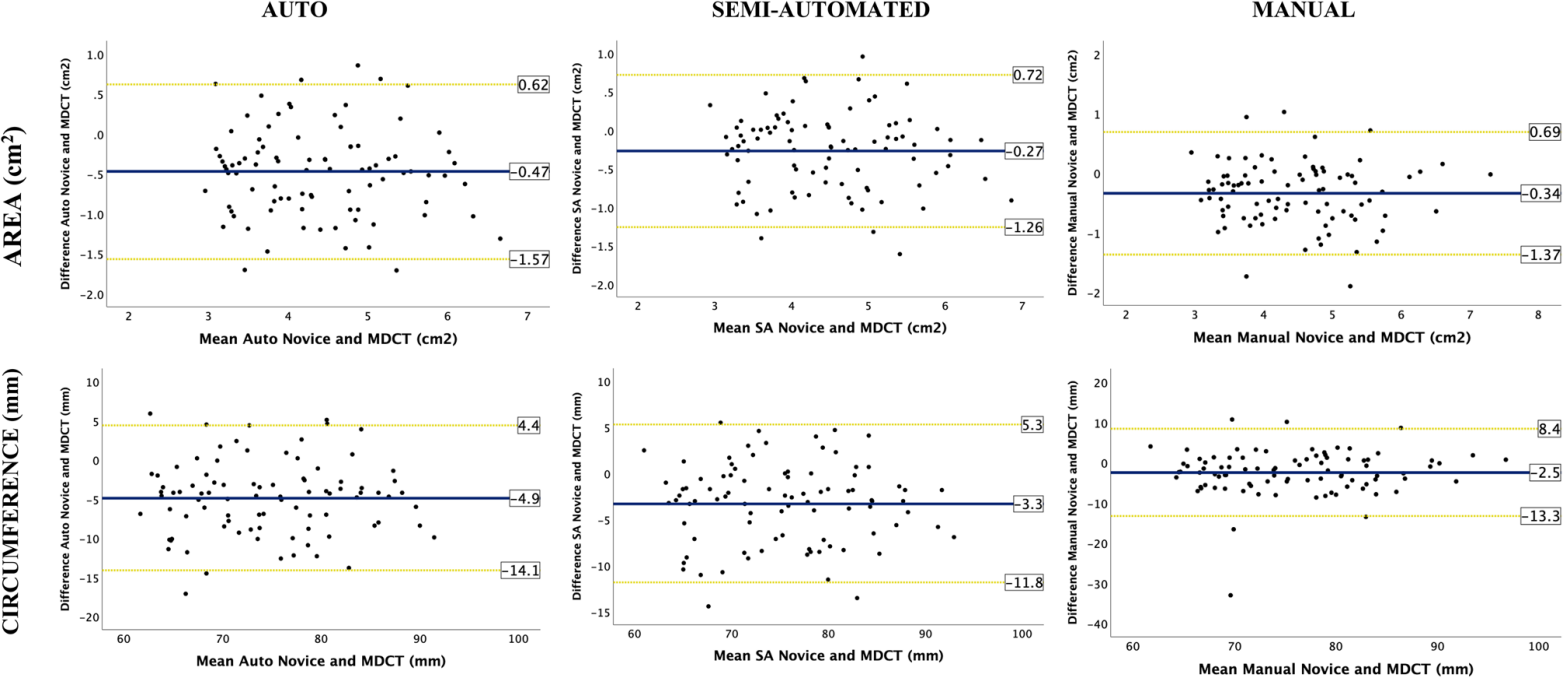
TEE novice vs. MDCT The Bland–Altman plots for aortic annular area (AAA) and circumference by automated, semi-automated and manual methods done by novice compared to MDCT

For the novice operator, good agreement was found between all three TEE methods and MDCT (Table 3). The SA method offered the smallest systematic bias for AAA and overall narrower LOA compared to MDCT for AAA and circumference among the three methods (Table 3, Fig. 3). The auto method had the largest bias for AAA and circumference.

#### Correlation

There is very good correlation with MDCT for all 3 methods (r spans from 0.78 to 0.86) with the SA method offering the best correlation overall and the manual method the least robust one (Table S4).

### (C) Novice TEE vs. expert TEE

**Fig. 4:**
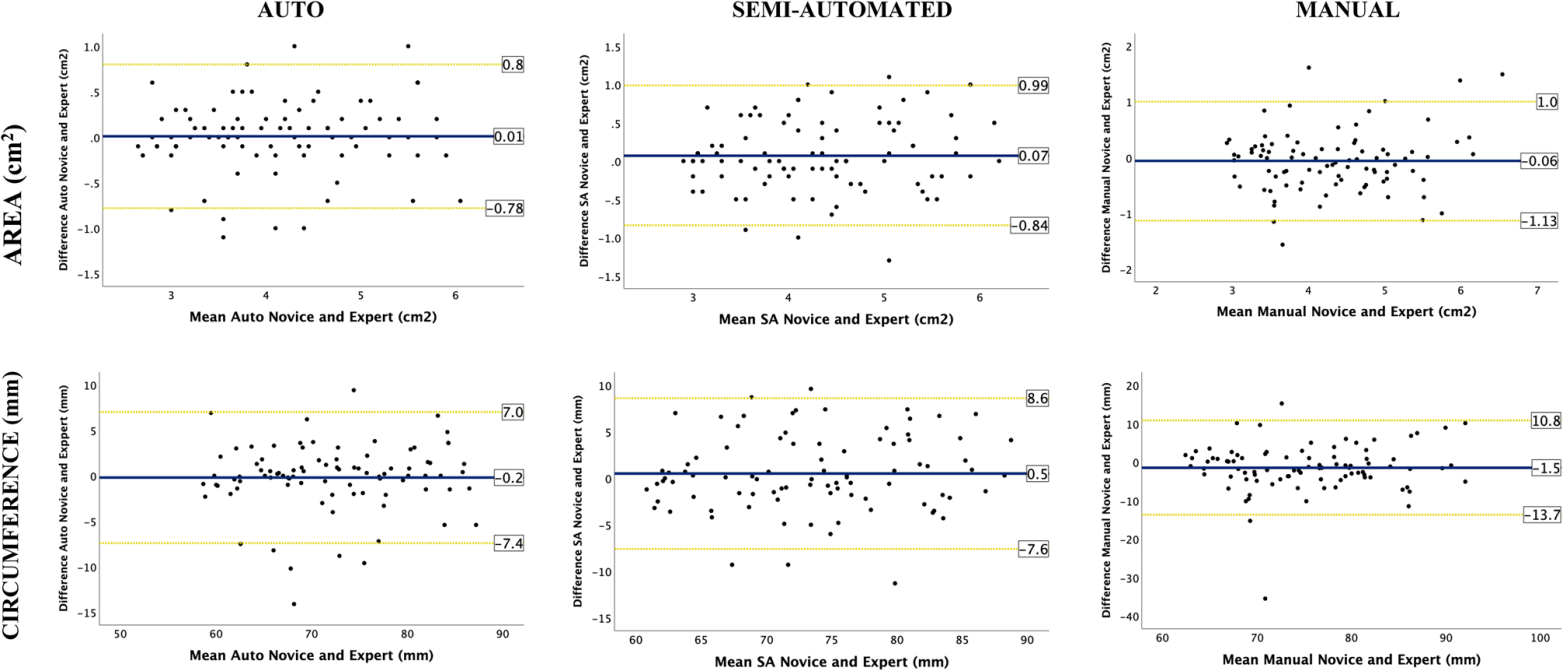
TEE novice vs. TEE expert The Bland–Altman plots for aortic annular area and circumference by automated, semi-automated and manual methods done by novice compared to expert 3D-TEE measurements

When comparing novice to expert measurements, the auto method had the smallest systematic bias and narrower LOA among all methods for AAA and circumference (Table 3). Overall, there is good agreement between novice and experts for all 3 methods, but better agreement with auto and SA methods (Table 3, Fig. 4). Auto and SA methods offer very good correlation to expert measurements (r spans from 0.86 to 0.91), as opposed to manual method which only offers good correlation with experts (r from 0.71 to 0.82) (Table S4).

### (D) Reproducibility

Analysis of intra- and inter-observer variability for the auto, SA, and manual methods in 35 patients demonstrated excellent agreement between observations for experts (intra-observer ICC range from 0.98 to 0.99; inter-observer ICC range from 0.96 to 0.97) (Table S5). Intra-observer reproducibility in novice was excellent for the auto and SA methods (intra-observer ICC 0.98 to 0.99), and good for the manual method (ICC 0.82 to 0.83) (Table S6). Intra-observer reproducibility for MDCT measurements was excellent (ICC 0.98) (Table S7).

### (E) Agreement with final prosthesis size

**Fig. 5:**
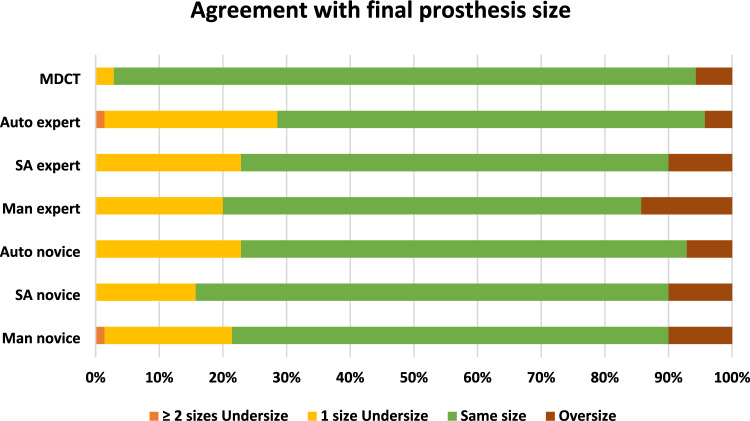
Agreement between hypothetical prosthesis size derived from 3D-TEE and MDCT and final size of implanted prosthesis Auto expert = automated method by expert; SA expert = semi-automated method by expert; Man expert = manual method by expert; Auto novice = automated method by novice; SA novice = semi-automated method by novice; Man novice = manual method by novice

Hypothetical valve sizing using 3D-TEE measurements (independently of the method used) and manufacturer-generated algorithms agreed with the final prosthesis size in 69% of cases on average vs. 91% of cases for MDCT (Fig. 5, Table S8) and undersized the prosthesis in 22% of cases. Concordance with final prosthesis size using 3D-TEE was obtained most frequently using the SA and auto methods for experts (67% concordance) and the SA method for novice (74%).

## Discussion

The present study is the most comprehensive assessment of the newer GE automatic 3D software for AoA sizing (GE AVQ) as it includes expert and novice readers and reports for the first time the performance of the fully automated model with no manual editing. It demonstrates that:

1. TEE measurements with auto, SA and manual methods have good to very good agreement and correlation with the reference MDCT measurements.
2. The software-derived auto method tends to underestimate the most AoA dimensions vs. MDCT both for experts and novice operators.
3. For expert operators, the manual TEE method still offers the least bias vs. MDCT, but SA and auto methods have narrower LOA and slightly better correlation.
4. For a novice operator, SA appears to be the method of choice as it provides less bias, narrower LOA, and better correlation with MDCT.
5. There is good agreement between novice and experts for all three TEE methods but better agreement and correlation with auto and SA methods as opposed to manual one.
6. All three TEE methods are reproducible for AoA sizing in experts and novice but auto and SA methods show better intraobserver reproducibility in novices.
7. Hypothetical prosthesis sizing using 3D-TEE leads to moderate agreement with final prosthesis size implanted.

### 3D-TEE as alternative to MDCT

Several studies including a meta-analysis [21] have now shown 3D-TEE to be a feasible alternative to MDCT for aortic annulus sizing [12, 14, 21, 22]. Our study adds to this growing body of evidence with 3D-TEE measurements offering good to very good agreement and correlation with MDCT values, independently of the method used. This includes the fully automated model for blood-tissue border tracing that requires minimal operator training, intervention, and editing.

AoA sizing remains a challenging and essential step of pre-TAVI planning. Both MDCT and 3D-TEE methods involve multiplane reformatting and active manual contouring of the annulus at the blood-tissue interface with LVOT calcifications complicating measurements, even for expert operators. Our study is consistent with previous reports of annulus undersizing with TEE. The SA method undersized AAA on average by 6.5% in experts and novices. The manual method showed a non-significant difference in circumference to MDCT in experts and a 3.2% difference in novices. This systematic underestimation is lower than the 9–20% undersizing found in initial studies [11, 22–24], but greater than the small absolute difference (< 1%) reported by Khalique et al. [22] and the non-significant differences reported in a meta-analysis of 1599 patients [21]. The magnitude of the percent change observed remains within acceptable inter- and intraobserver variability for TEE and MDCT-based measurements and may not translate to adverse clinical outcomes.

### Automated, SA vs. manual 3D-TEE methods

Contemporary automated 3D reformatting methods using an off-label mitral valve software (Philips Q-lab MVQ) [22, 25] and more recently different software dedicated to AoA sizing (Philips Aortic Valve Navigator [14], Philips Q-lab [26], Speqle3D [13], and Auto AVQ GE [12]) showed excellent correlation to MDCT and a lower bias, in the range of 1–2%.

The GE Auto AVQ software, which is particularly intuitive (requiring less than 30 seconds when fully automated, and minimal input from the operator) was shown to have attractive test characteristics being reproducible, highly correlative to MDCT and performing well irrespective of the presence of LVOT calcifications [12] In the current study, we have performed the most comprehensive assessment of the GE Auto AVQ software in both expert and novice readers, reporting for the first time the performance of the fully automated method in which manual editing was not allowed, in addition to the SA (manual editing allowed) and manual methods. Our study shows that each method bears its advantages and disadvantages.

The fully automated method, while still maintaining overall good agreement with MDCT, is the TEE method that numerically underestimates the most AoA dimensions both in experts and novices vs. MDCT. The software, which tends to draw the borders of the annulus towards the inside of the lumen, is possibly confounded by a hazy blood-tissue interface that is blurred by the calcified LVOT walls. However, of all TEE methods it showed very good correlation with MDCT, is the most reproducible independently of the operator’s experience and has the best agreement and correlation between novice and experts.

Interestingly, manual measurements of AAA and circumference done by experts had the smallest bias vs. MDCT when compared to other TEE methods, but slightly lower correlation, precision, and reproducibility. The manual method involves a turn-around technique in which orthogonal cutting planes are rotated along each leaflet to identify the leaflets hinge points. This careful contouring of the true annulus is unique to the manual method and mimics the multiplanar reformations done on a MDCT dataset [9]. Moreover, expert operators have a better understanding of tissue-lumen interface in the presence of significant calcifications, which is not always adequately appreciated by automatically generated algorithms, as we have shown.

### Expert vs. novice

In a novice operator, the SA method appears to be the method of choice, as it has the least bias, narrower LOA, and best correlation with MDCT overall. This is in keeping with the results of Khalique et al. [25] who showed that a novice (cardiologist without significant experience in 3D echocardiography) had better correlation and narrower LOA for AoA sizing using the off-label MVQ (Mitral Valve Quantification) software than with manual direct planimetry. While there is still a role for manual AoA sizing in experienced operators, it appears to be a less appealing option for novices. Auto and SA methods, which were shown in our study to be more reproducible, hence less operator-dependent, allow novice operators to approximate TEE values obtained by experts as well as MDCT values. This will potentially be beneficial in low and intermediate-volume TAVI centers as well as referring centers who might have less experience with TEE-guided AoA sizing, and in cases where renal failure or a severe iodinated contrast allergy precludes the use of MDCT altogether.

Overall, the auto method is easy to use, reproducible and brings the novice closer to the expert but at the cost of greater AoA underestimation. The SA method helps the novice to approximate MDCT measurements and is a precise method for experts and novices alike. Finally, the manual TEE method has better agreement vs. MDCT in experts but is less precise, more time-consuming and has a steeper learning curve, making it less well adapted to novice readers.

### Hypothetical prosthesis sizing

Hypothetical prosthesis sizing using 3D-TEE showed 69% agreement with final prosthesis size and 22% valve undersizing. This is consistent with the report from Stella et al., whereby a 3.5% underestimation of AAA with TEE was associated with only 65% agreement with final prosthesis size [12]. This contrasts with an earlier report [25] in which a similar 3.2% underestimation of AAA led to valve size agreement in 94 cases out of 100. The fact that MDCT-derived hypothetical sizing was not 100% concordant with the valve implanted reflects that prosthesis selection is a complex and multifactorial decision that integrates multiples factors besides AoA size including presence of extensive LVOT calcifications, vascular access, elliptical annulus and risk of permanent conduction abnormalities to name a few [27]. This reinforces the importance of multimodality imaging in aortic annulus sizing, if not in all patients, at least in those where MDCT is contraindicated or equivocal (e.g., presence of artefacts or borderline between sizes) in order to minimize adverse events, including significant PVR. Future iterations of the auto AVQ software involving advanced machine learning algorithms may be better suited to avoid this systematic underestimation.

## Limitations

Several limitations must be considered when interpreting the results of our study. First, this was a small, single center cohort study. Validation in a different and larger population, ideally multicentric, would reinforce our findings. However, there was excellent inter- and intra-observer reproducibility of the measurements, and our results are consistent with previous reports with regards to the range of annulus undersizing. The automatic software does not allow us to measure surrounding structures such as coronary height and aortic root diameter. However, standard 2D-TTE and TEE are usually sufficient to estimate those variables, which are less critical to procedural planning. No analyses of the discriminative ability of different imaging modalities to predict prosthesis size and post-procedure PVR was performed, as the incidence of significant PVR was low in our study. No inter-observer variability analyses were done for MDCT. Finally, the novice operator acquired experience during the data collection process which may have taken his skills beyond those of a true novice and potentially influenced the results of our study.

## Conclusion

Our study supports the use of 3D-TEE as a complementary method to MDCT for aortic annular sizing in preparation for TAVI. The newer automated and semi-automated 3D-TEE software, 4D Auto AVQ, is an easy to use, reproducible and reliable option that can help bridge the gap between expert operators and less experienced ones.

### Supplementary Information

Below is the link to the electronic supplementary material.

Supplementary file1 (DOCX 45567 KB)

## Abbreviations

3D-TEE: Three-dimensional transesophageal echocardiography
AoA: Aortic annulus
AAA: Aortic annular area
AVQ: Aortic valve quantification
Dmax: Maximal diameter
Dmean: Mean diameter
Dmin: Minimal diameter
ICC: Intraclass correlation coefficient
LOA: Limits of agreement
LVOT: Left ventricular outflow tract
MDCT: Multidetector row computed tomography
PVR: Paravalvular regurgitation
SA: Semi-automated
TAVI: Transcatheter aortic valve implantation
