# Supplementary material for: Automated and semi-automated 3D echocardiographic software for aortic annulus sizing in transcatheter aortic valve implantation helps bridge the gap between expert and novice operators
Source: Int J Cardiovasc Imaging. 2023 Aug 12;39(9):1707–17. doi: 10.1007/s10554-023-02885-z (PMC10520092; doi:10.1007/s10554-023-02885-z)
Supplement: Supplementary file 1 — Supplementary file1 (DOCX 45567 KB) [file 10554_2023_2885_MOESM1_ESM.docx]

**Automated and Semi-Automated 3D Echocardiographic Software for Aortic Annulus Sizing in Transcatheter Aortic Valve Implantation helps bridge the gap between expert and novice operators**

Charles Massie, Martine Parent, Maxime Berthelot-Richer, Rémi Kouz, Donald Palisaitis, Viet Le, Frédéric Poulin.

**The International Journal of Cardiovascular Imaging**

Author of correspondence:

Frédéric Poulin, MD, MSc

Hôpital du Sacré-Cœur, 5400 Boul Gouin O, H4J 1C5

f.poulin@umontreal.ca

**SUPPLEMENTAL APPENDIX**

**Table S1 TAVI procedural outcomes (n = 70)**

| **TAVI adverse outcomes N (%)** | | |
| --- | --- | --- |
| Ischemic stroke |  | 5 (7) |
| Intracranial hematoma |  | 0 (0) |
| Femoral bleed |  | 14 (20) |
| Aortic root injury or rupture |  | 0 (0) |
| New onset LBBB |  | 12 (17) |
| New onset 3rd degree AVB |  | 8 (11) |
| Pacemaker implantation |  | 11 (16) |
| Perioperative mortality |  | 0 (0) |
| PVR post-op day 1 (TTE) |  |  |
| none |  | 33 (47) |
| trace |  | 25 (36) |
| mild |  | 10 (14) |
| mild to moderate |  | 2 (3) |
| moderate |  | 0 (0) |
| severe |  | 0 (0) |
| PVR 1-month post-op (TTE) |  |  |
| none |  | 25 (37) |
| trace |  | 22 (33) |
| mild |  | 16 (24) |
| mild to moderate |  | 3 (4) |
| moderate |  | 1 (1) |
| severe |  | 0 (0) |
| Mean prosthetic gradient 1-month post-op, mmHg. |  | 9.7 ± 5.1 |
| Values are mean ± SD or frequencies (percentage).  LBBB = left bundle branch block; AVB = Atrioventricular block; PVR = paravalvular regurgitation; TTE = transthoracic echocardiography. | | |

**Table S2 Additional aortic annular measurements derived from different imaging methods (n=89)**

|  | EXPERT | | | NOVICE | | | MDCT |
| --- | --- | --- | --- | --- | --- | --- | --- |
|  | **auto** | **semi-automatic** | **manual** | **auto** | **semi-automatic** | **manual** |  |
| **Dmax (mm)** | 25.2 ± 3.0* | 25.0 ± 2.7* | 25.8 ± 3.0* | 24.6 ± 2.8*† | 25.0 ± 2.8* | 24.1 ± 2.8*† | 27.0 ± 3.0 |
| **Dmin (mm)** | 20.5 ± 2.6* | 21.5 ± 2.3 | 21.0 ± 2.3* | 21.2 ± 2.7† | 21.9 ± 2.5† | 21.6 ± 2.6† | 21.7 ± 2.4 |
| **Dmean (mm)** | 23.0 ± 2.5* | 23.3 ± 2.4* | 23.4 ± 2.4* | 22.9 ± 2.6* | 23.5 ± 2.5* | 22.9 ± 2.4*† | 24.3 ± 2.5 |

Dmax = maximal diameter of the aortic annulus; Dmin = minimal diameter of the aortic annulus; Dmean = mean diameter of the aortic annulus; auto = automated method, MDCT = multidetector row computed tomography.

* p ˂0.001 for the comparisons between the TEE measurement vs. the reference MDCT.

† p ˂0.05 for the comparisons between the TEE measurement by novice vs. expert.

**Table S3 Average bias and LOA between modalities – Dmean, Dmax and Dmin**

|  | **TTE expert vs. MDCT** | | | | | | **TEE novice vs. MDCT** | | | | | | | **TEE novice vs. TEE expert** | | | | | |  |
| --- | --- | --- | --- | --- | --- | --- | --- | --- | --- | --- | --- | --- | --- | --- | --- | --- | --- | --- | --- | --- |
|  | **Auto** | | **SA** | | **Manual** | | **Auto** | | | **SA** | | **Manual** | | **Auto** | | **SA** | | **Manual** | |  |
|  | **MD ± SD** | **± 1.96 SD** | **MD ± SD** | **± 1.96 SD** | **MD ± SD** | **± 1.96 SD** | **MD ± SD** | | **± 1.96 SD** | **MD ± SD** | **± 1.96 SD** | **MD ± SD** | **± 1.96 SD** | **MD ± SD** | **± 1.96 SD** | **MD ± SD** | **± 1.96 SD** | **MD ± SD** | **± 1.96 SD** |  |
| **Dmean** | -1.3 ± 1.5 | 5.8 | -1.0 ± 1.5 | 5.8 | -0.9 ± 1.8 | 6.9 | -1.4 ± 1.5 | | 5.9 | -0.8 ± 1.4 | 5.6 | -1.5 ± 1.5 | 5.8 | -0.1 ± 1.1 | 4.4 | 0.2 ± 1.3 | 5.2 | -0.6 ± 1.9 | 7.6 |  |
| **Dmax** | -1.8 ± 2.4 | 9.3 | -2.0 ± 2.1 | 8.2 | -1.2 ±2.5 | 10.1 | -2.3 ± 2 | | 7.8 | -2.0 ± 2.0 | 7.9 | -2.9 ±2.3 | 8.8 | -0.6 ± 1.8 | 7.1 | -0.04 ± 2.0 | 7.7 | -1.7 ± 2.7 | 10.7 |  |
| **Dmin** | -1.1 ± 2.0 | 7.6 | -0.1 ± 1.6 | 6.2 | -0.6 ±1.7 | 6.6 | -0.5 ± 1.9 | | 7.5 | 0.2 ± 1.7 | 6.7 | -0.1 ± 1.8 | 7.0 | 0.6 ± 1.8 | 7.0 | 0.4 ±1.2 | 4.7 | 0.5 ± 2.2 | 8.7 |  |
|  |  |  |  |  |  |  |  |  |  |  |  |  |  |  |  |  |  |  |  |  |

LOA = limits of agreement; Dmean = mean diameter of the aortic annulus; Dmax = maximal diameter of the aortic annulus; Dmin, minimal diameter of the aortic annulus; MD = mean difference; SD = standard deviation; Auto = automated method; SA = semi-automated method; TEE expert = transesophageal echocardiography done by expert operator; TEE novice = transesophageal echocardiography done by novice operator; MDCT = multidetector computed tomography.

**Table S4 Correlation between modalities**

|  | **TEE expert vs. MDCT** | | | **TEE novice vs. MDCT** | | | **TEE novice vs. TEE expert** | | |
| --- | --- | --- | --- | --- | --- | --- | --- | --- | --- |
|  | **Auto** | **SA** | **Manual** | **Auto** | **SA** | **Manual** | **Auto** | **SA** | **Manual** |
| **AAA** | 0.83  (0.77-0.89) | 0.82  (0.74-0.88) | 0.82  (0.76-0.88) | 0.83  (0.77-0.88) | 0.86  (0.80-0.91) | 0.85  (0.78-0.90) | 0.91  (0.86-0.94) | 0.87  (0.81-0.91) | 0.82  (0.75-0.88) |
| **Circumference** | 0.83  (0.77-0.88) | 0.83  (0.76-0.88) | 0.81  (0.73-0.87) | 0.83  (0.77-0.88) | 0.85  (0.80-0.90) | 0.78  (0.60-0.89) | 0.90  (0.84-0.94) | 0.86  (0.80-0.91) | 0.71  (0.51-0.85) |

P < 0.001 for all; data presented as: correlation coefficient *r* (bootstrap 95% confidence interval).

AAA = aortic annular area; Auto = automated method; SA = semi-automated method; TEE expert = transesophageal echocardiography done by expert operator; TEE novice = transesophageal echocardiography done by novice operator; MDCT = multidetector row computed tomography.

**Table S5 Inter- and intra-observer variability for 3D-TEE expert measurements**

|  | **Inter-observer (n=35)** | | | | | | **Intra-observer (n=35)** | | | | | |
| --- | --- | --- | --- | --- | --- | --- | --- | --- | --- | --- | --- | --- |
|  | **Auto** | | **SA** | | **Manual** | | **Auto** | | **SA** | | **Manual** | |
|  | ICC | 95% CI | ICC | 95% CI | ICC | 95% CI | ICC | 95% CI | ICC | 95% CI | ICC | 95% CI |
| **AAA** | 0.97 | 0.94-0.98 | 0.97 | 0.94-0.99 | 0.96 | 0.92-0.98 | 0.99 | 0.99-1.00 | 0.98 | 0.96-0.99 | 0.98 | 0.96-0.99 |
| **Circumference** | 0.97 | 0.94-0.99 | 0.97 | 0.93-0.98 | 0.96 | 0.92-0.98 | 0.99 | 0.99-1.00 | 0.98 | 0.96-0.99 | 0.98 | 0.96-0.99 |

AAA = aortic annular area; ICC = intraclass correlation coefficient; 95% CI = 95% confidence interval; Auto = automated method; SA = semi-automated method.

**Table S6 Intra-observer variability for 3D-TEE novice measurements**

|  | **Intra-observer (n=20)** | | | | | |
| --- | --- | --- | --- | --- | --- | --- |
|  | **Auto** | | **SA** | | **Manual** | |
|  | ICC | 95% CI | ICC | 95% CI | ICC | 95% CI |
| **AAA** | 0.99 | 0.98-1.00 | 0.98 | 0.95-0.99 | 0.83 | 0.56-0.93 |
| **Circumference** | 0.99 | 0.98-1.00 | 0.98 | 0.94-0.99 | 0.82 | 0.54-0.93 |

AAA = aortic annular area; ICC = intraclass correlation coefficient; 95% CI = 95% confidence interval; Auto = automated method; SA = semi-automated method.

**Table S7 Intra-observer variability for MDCT measurements**

|  | **Intra-observer (n=20)** | |
| --- | --- | --- |
|  | **MDCT** | |
|  | ICC | 95% CI |
| **AAA** | 0.98 | 0.94-0.99 |
| **Circumference** | 0.98 | 0.94-0.99 |

AAA = aortic annular area; ICC = intraclass correlation coefficient; 95% CI = 95% confidence interval; MDCT = multidetector computed tomography.

**Table S8 Agreement between hypothetical prosthesis size derived from 3D-TEE and MDCT and final size of implanted prosthesis**

|  | **EXPERT** | | | **NOVICE** | | | **MDCT** |
| --- | --- | --- | --- | --- | --- | --- | --- |
|  | **Auto** | **SA** | **Manual** | **Auto** | **SA** | **Manual** |  |
| 1 size oversize | 3 (4) | 7 (10) | 10 (14) | 5 (7) | 7 (10) | 7 (10) | 4 (6) |
| Concordant size | 47 (67) | 47 (67) | 46 (66) | 49 (70) | 52 (74) | 48 (69) | 64 (91) |
| 1 size undersize | 19 (27) | 16 (23) | 14 (20) | 16 (23) | 11 (16) | 14 (20) | 2 (3) |
| 2 sizes undersize | 1 (1) | 0 (0) | 0 (0) | 0 (0) | 0 (0) | 1 (1) | 0 (0) |

1 size oversize = oversizing of hypothetical valve by 1 size vs. final prosthesis size; Concordant size = hypothetical prosthesis size is the same size as final prosthesis size; 1 size undersize = undersizing of hypothetical valve by 1 size vs. final prosthesis size; undersizing of hypothetical valve by 2 sizes or more vs. final prosthesis size; Auto = automated method; SA = semi-automated method; MDCT = multidetector computed tomography.

**Fig. S1 TTE expert vs. MDCT** The Bland-Altman plots for Dmean, Dmax and Dmin by automated, semi-automated and manual methods done by experts compared to MDCT. Dmean = mean diameter of the aortic annulus; Dmax = maximal diameter of the aortic annulus; Dmin = minimal diameter of the aortic annulus.

|  | **AUTO** | **SEMI-AUTOMATED** | **MANUAL** |
| --- | --- | --- | --- |
| **DMEAN (mm)** | **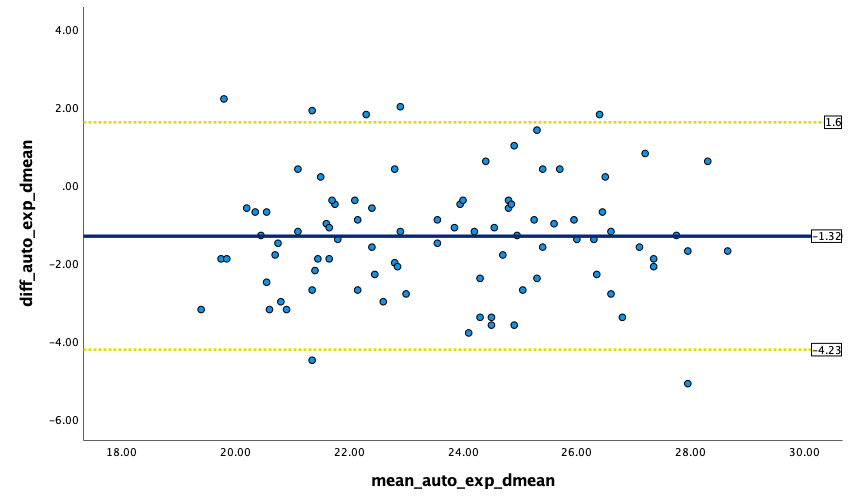** | **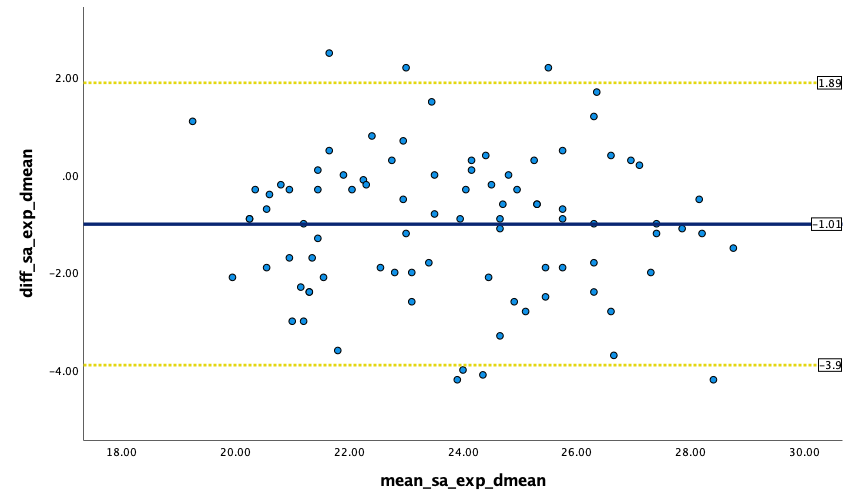** | **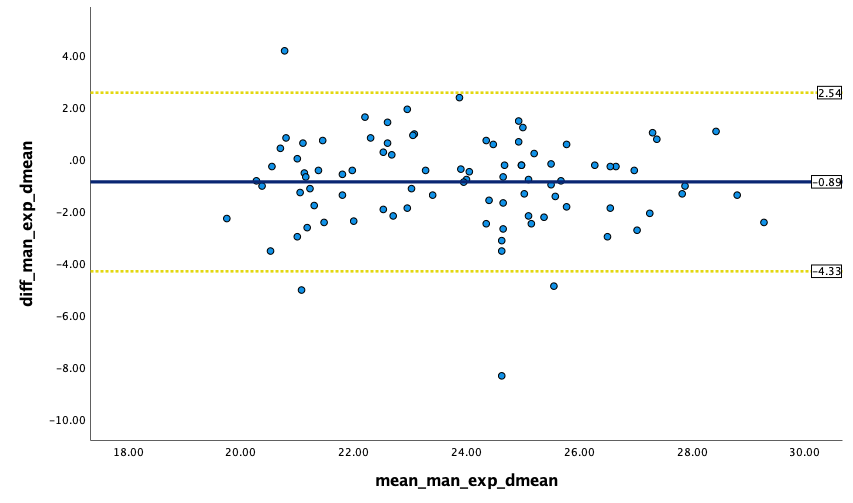** |
| **DMAX (mm)** | **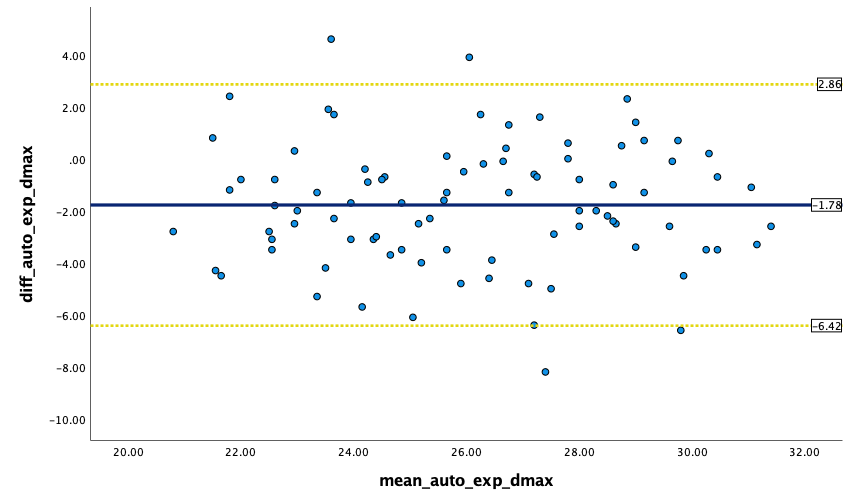** | **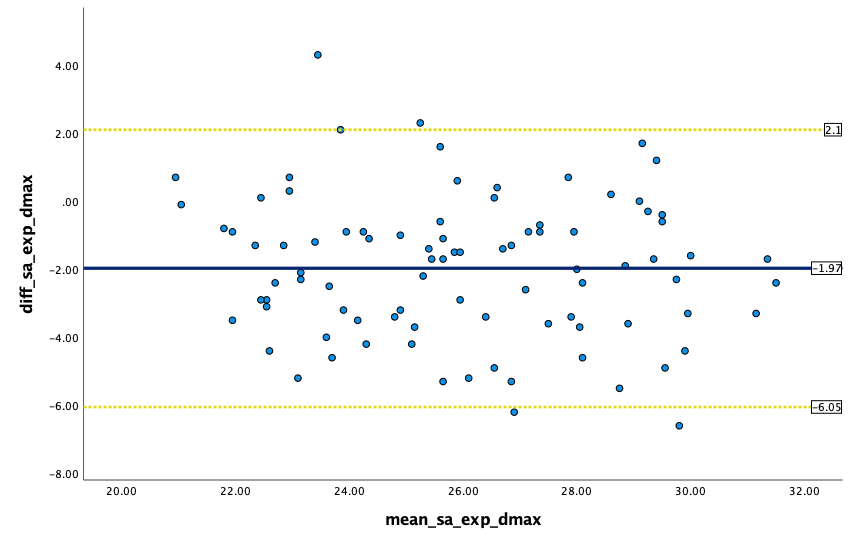** | **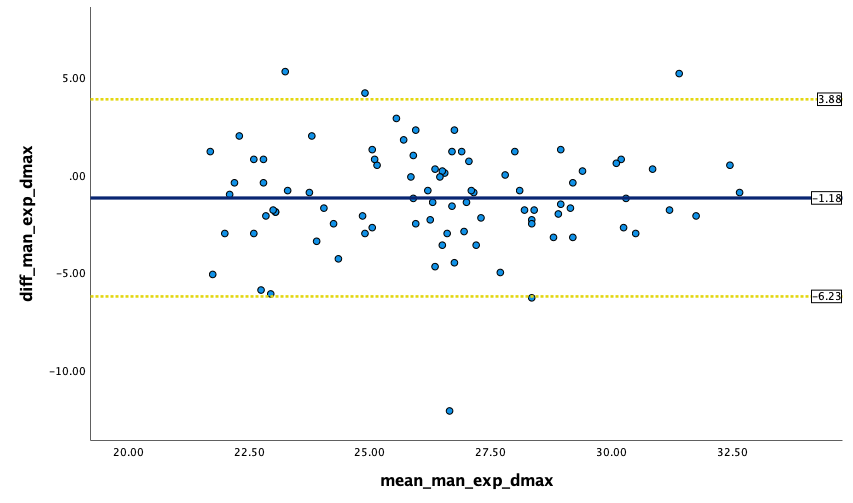** |
| **DMIN (mm)** | **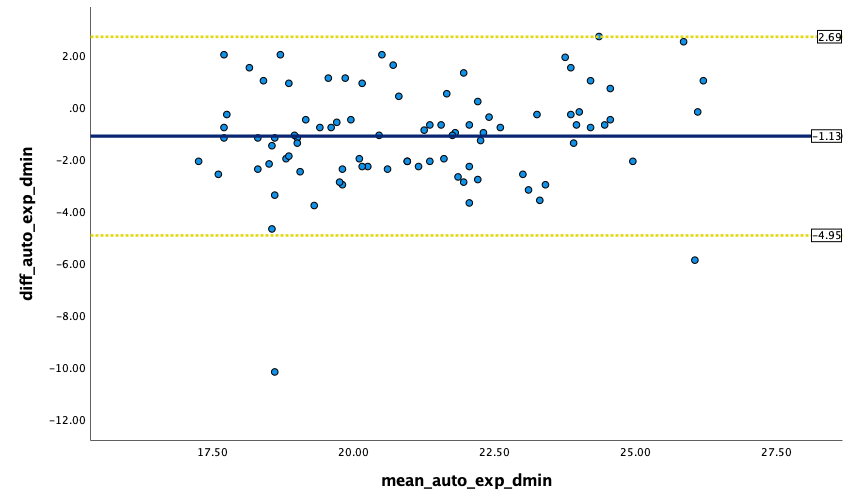** | **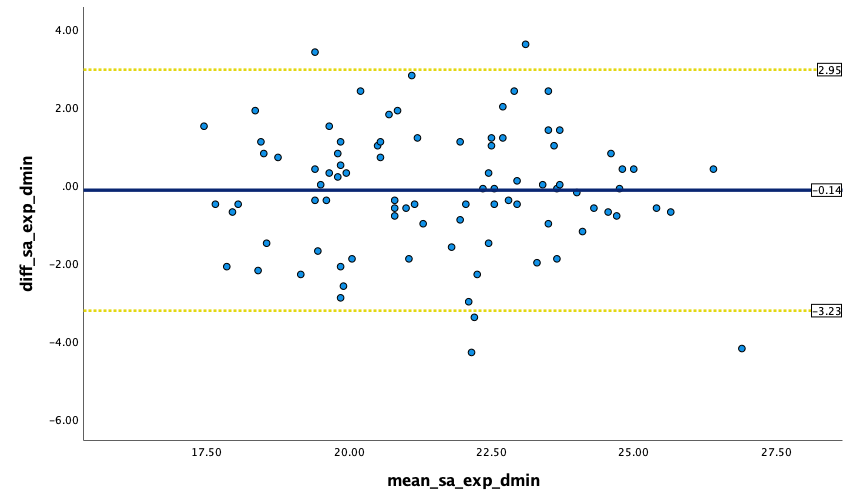** | **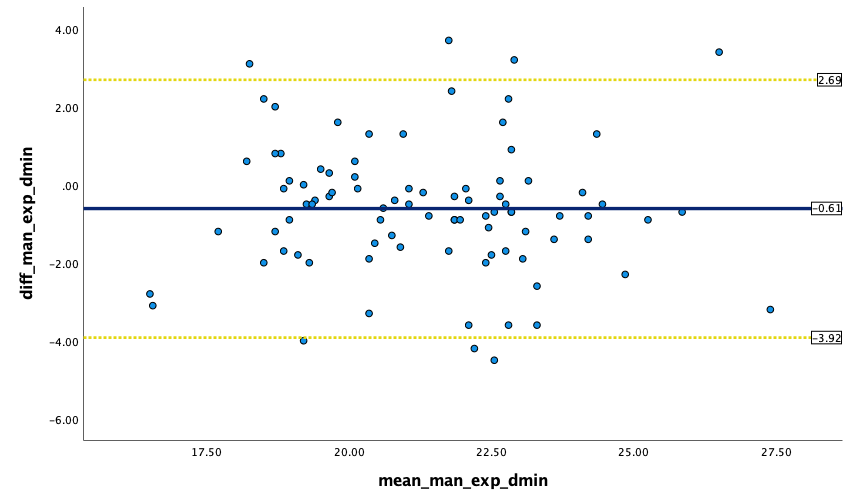** |

**Fig. S2 TTE novice vs. MDCT** The Bland-Altman plots for Dmean, Dmax and Dmin by automated, semi-automated and manual methods done by novice compared to MDCT. Dmean = mean diameter of the aortic annulus; Dmax = maximal diameter of the aortic annulus; Dmin = minimal diameter of the aortic annulus.

|  | **AUTO** | **SEMI-AUTOMATED** | **MANUAL** |
| --- | --- | --- | --- |
| **DMEAN (mm)** | **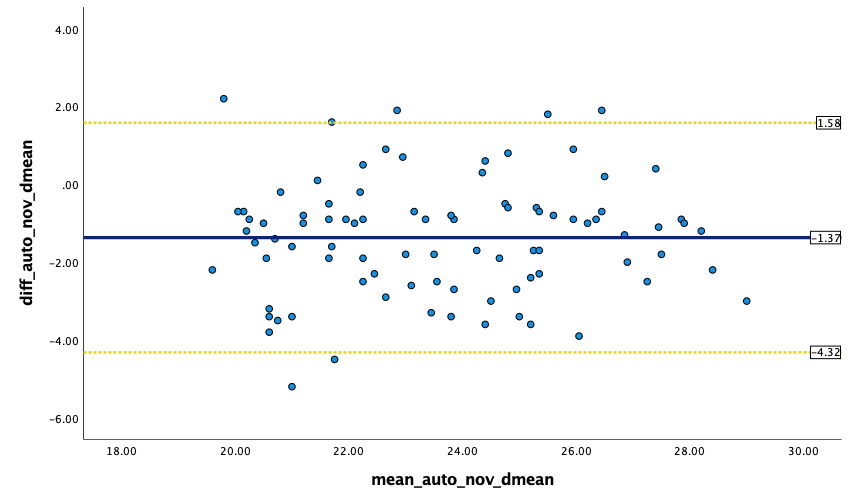** | 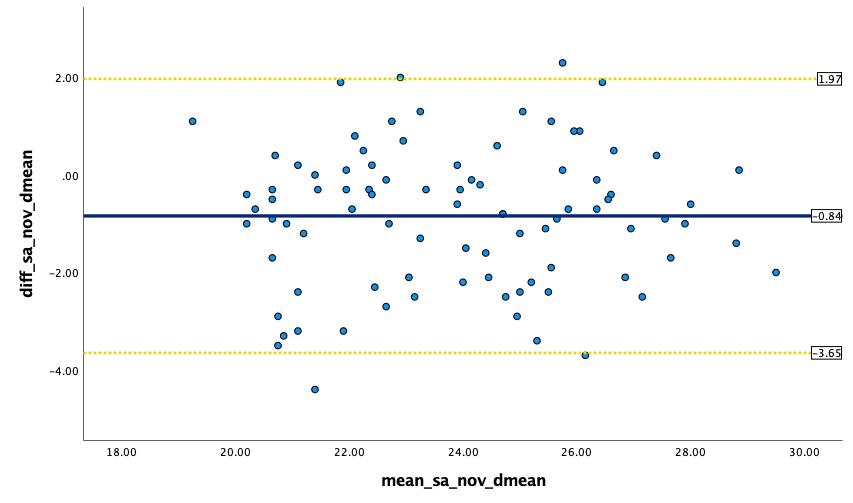 | 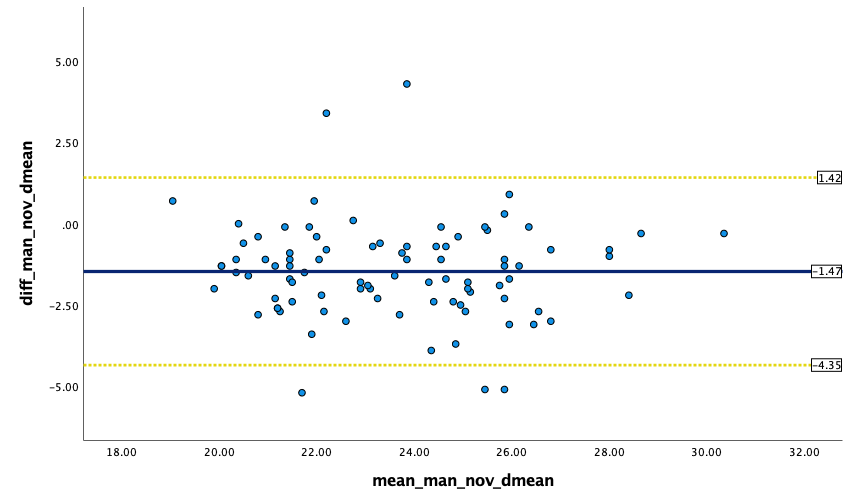 |
| **DMAX (mm)** | 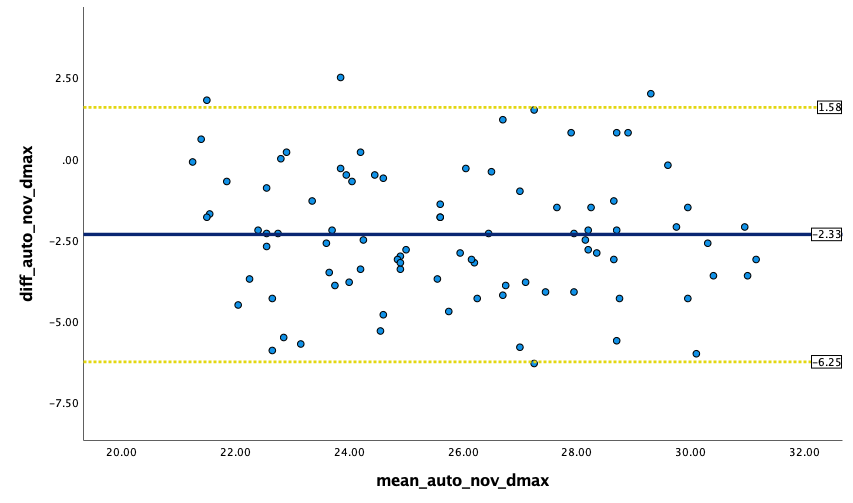 | 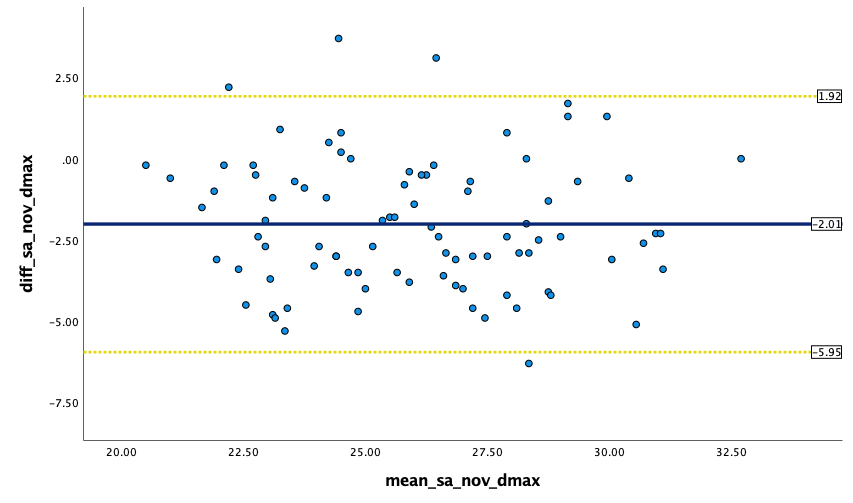 | 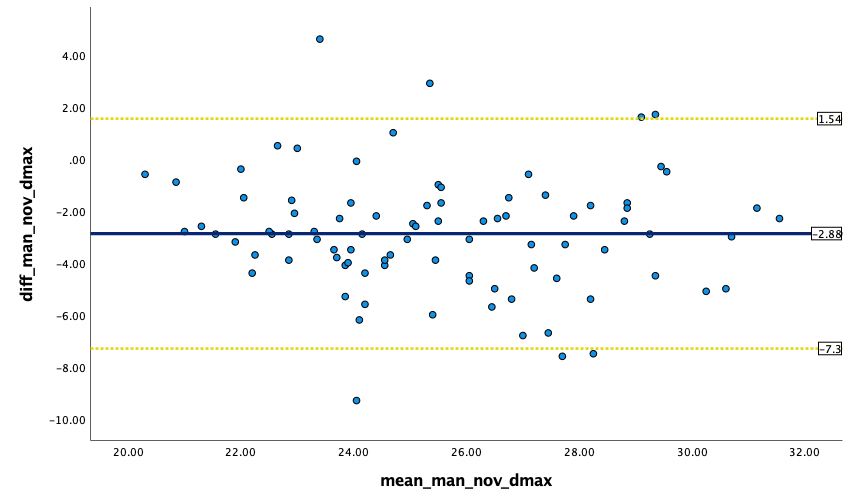 |
| **DMIN (mm)** | 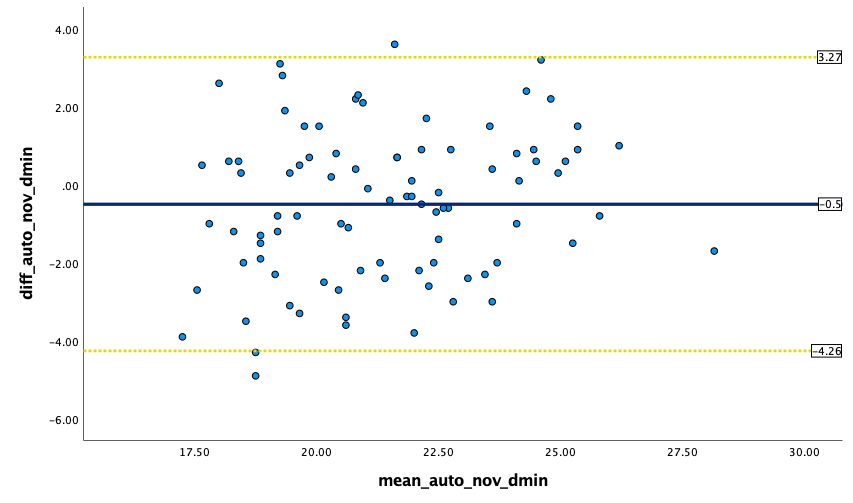 | 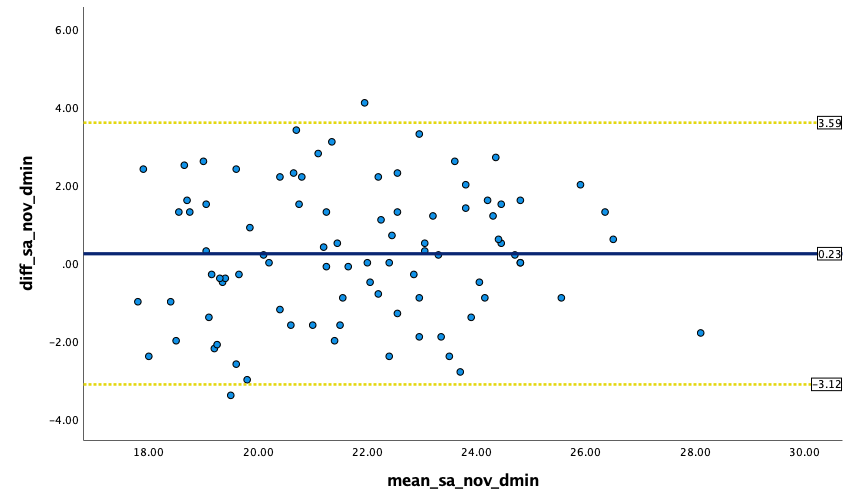 | 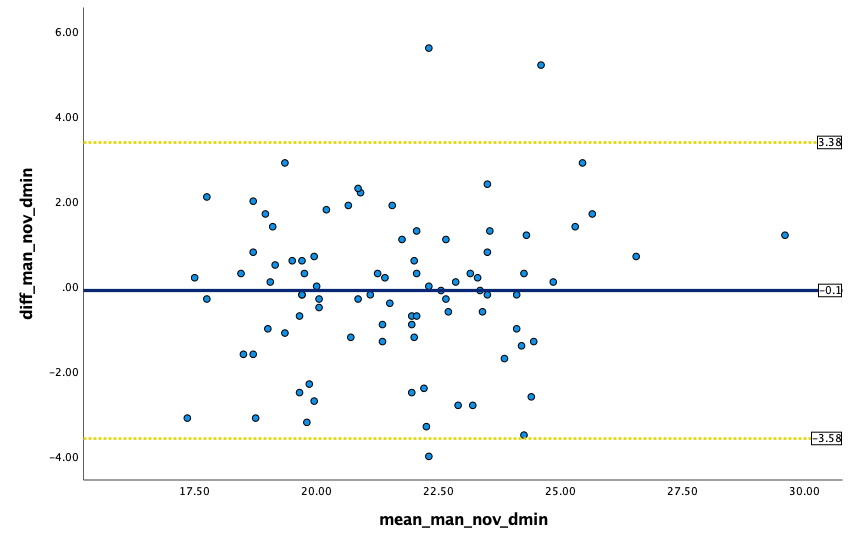 |

**Fig. S3 TTE novice vs. TTE expert** The Bland-Altman plots for Dmax, Dmax and Dmin by automated, semi-automated and manual methods done by novice compared to expert 3D-TEE measurements. Dmean = mean diameter of the aortic annulus; Dmax = maximal diameter of the aortic annulus; Dmin = minimal diameter of the aortic annulus.

|  | **AUTO** | **SEMI-AUTOMATED** | **MANUAL** |
| --- | --- | --- | --- |
| **DMEAN (mm)** | **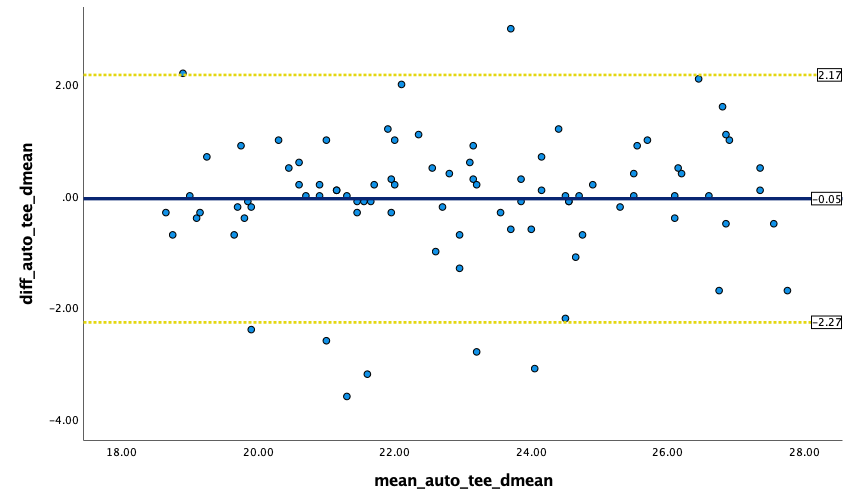** | 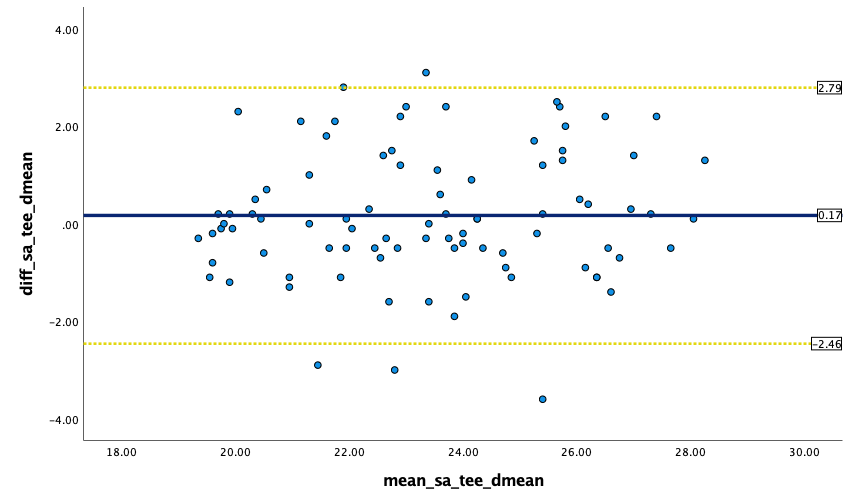 | 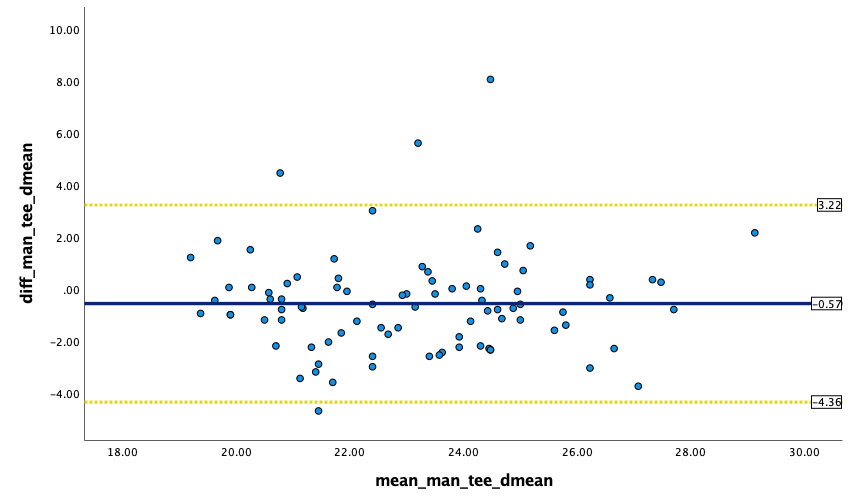 |
| **DMAX (mm)** | 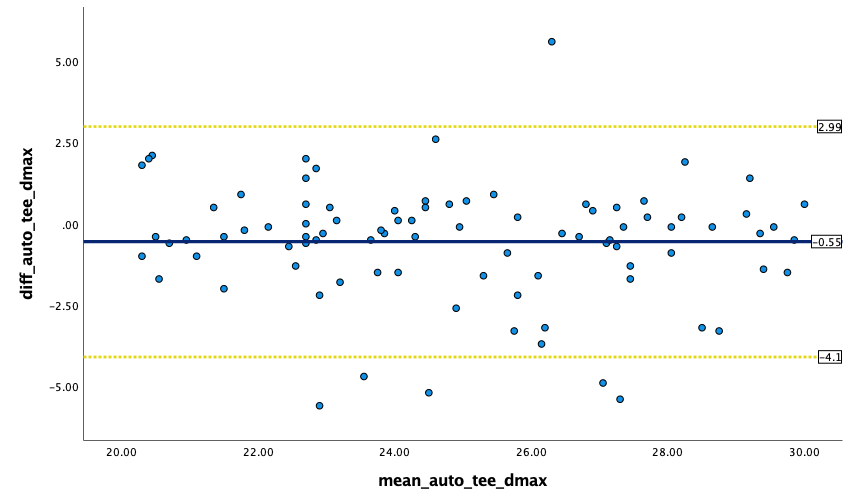 | 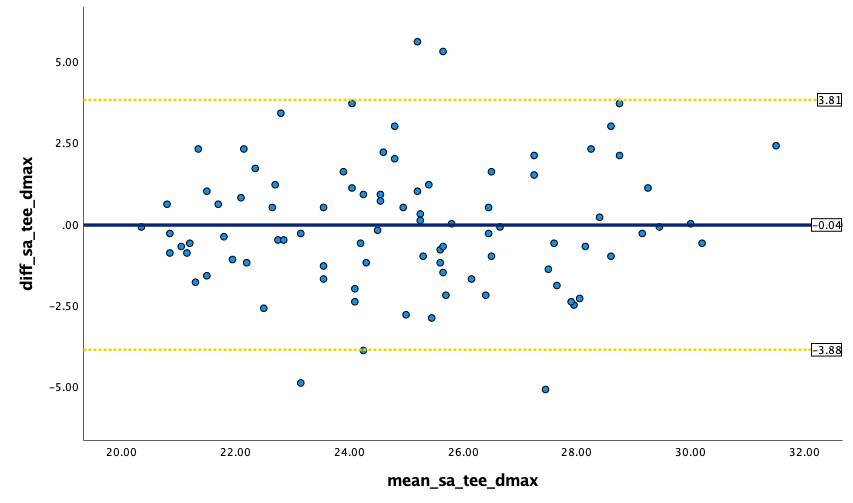 | 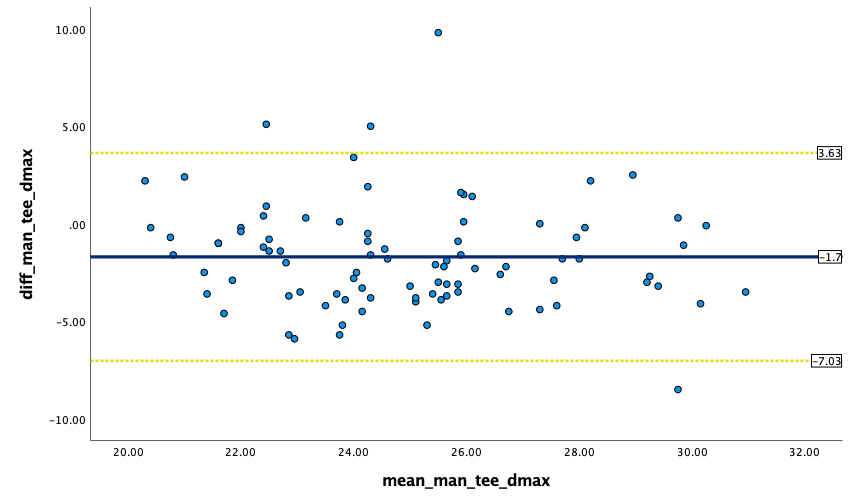 |
| **DMIN (mm)** | 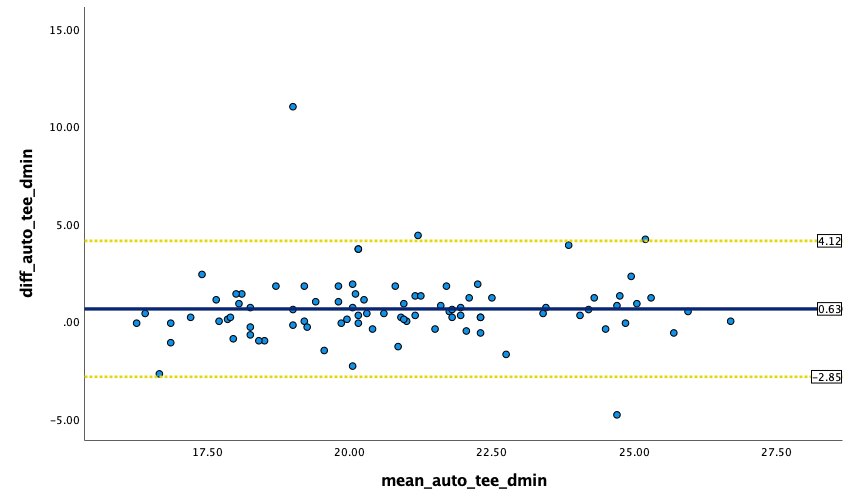 | 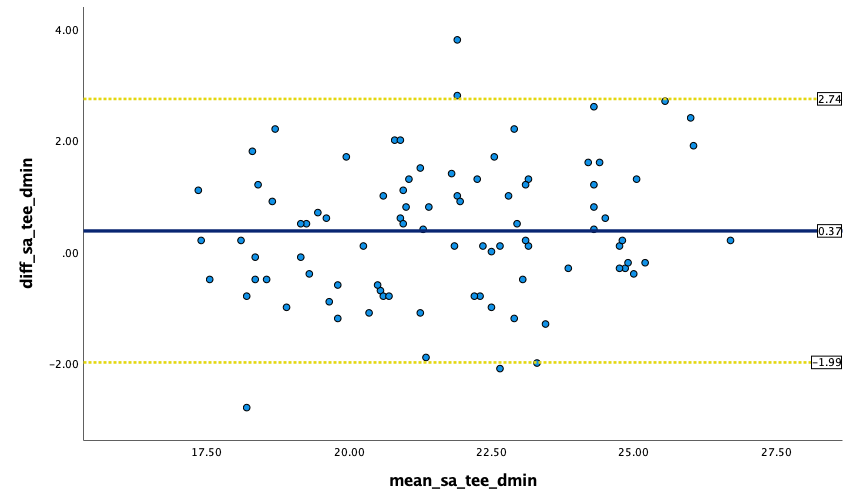 | 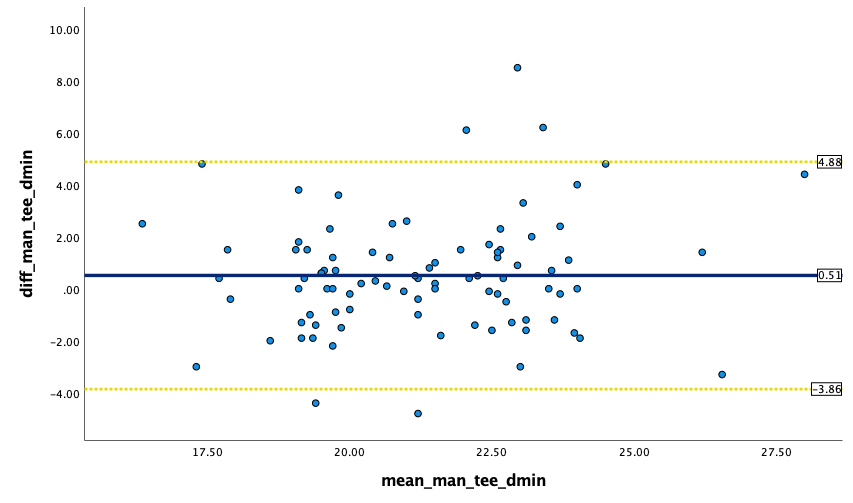 |
